# Germline Multigene Panel Testing in Women With Invasive Lobular Cancer

**DOI:** 10.1001/jamanetworkopen.2026.21705

**Published:** 2026-07-08

**Authors:** Giovanni Corso, Elena Marino, Francesca Fava, Fabrizio Natali, Paolo Peterlongo, Matteo Dal Molin, Irene Feroce, Cristina Zanzottera, Giulia Massari, Susanna Di Silvestre, Micol Moscatiello, Andrea Franceschini, Vincenzo Bagnardi, Giuseppe Curigliano, Bernardo Bonanni, Paolo Veronesi, Francesco Bertolini

**Affiliations:** 1 Division of Breast Surgery, European Institute of Oncology, IRCCS, Milan, Italy; 2 Department of Oncology and Hematology, University of Milano, Milan, Italy; 3 Laboratory of Hematology-Oncology, European Institute of Oncology, IRCCS, Milan, Italy; 4 Division of Cancer Prevention and Genetics, European Institute of Oncology, IRCCS, Milan, Italy; 5 Department of Statistics and Quantitative Methods, University of Milano-Bicocca, Milan, Italy; 6 School of Medicine and Surgery, University of Milano-Bicocca, Milan, Italy; 7 Division of New Drugs and Early Drug Development for Innovative Therapies, European Institute of Oncology, IRCCS, Milan, Italy; 8 Laboratory of Medical Genetics, Cytogenetics, and Molecular Genetics, European Institute of Oncology, IRCCS, Milan, Italy

## Abstract

**Importance:**

Invasive lobular carcinoma (ILC) represents the second most common histologic subtype of breast cancer (BC), yet its genomic landscape and clinical implications remain less well defined compared with invasive ductal carcinoma. Understanding genetic predisposition in ILC may improve risk assessment and guide tailored clinical management.

**Objectives:**

To investigate the prevalence and clinical outcomes of germline pathogenic or likely pathogenic variants (PVs) in BC predisposition genes among women with ILC and to assess the prognostic utility of polygenic risk scores (PRSs) in this population.

**Design, Setting, and Participants:**

This prospective, longitudinal cohort study was conducted at the European Institute of Oncology, Milan, Italy, from May 16, 2022, to January 31, 2025. Women diagnosed with primary ILC were enrolled and underwent multigene panel testing of 113 genes using next-generation sequencing. Follow-up data were collected until January 31, 2023. Statistical analysis was performed in January 2026.

**Main Outcomes and Measures:**

The primary outcome was BC-free survival, defined as the time from surgery to ipsilateral recurrence, contralateral disease, distant metastasis, or BC-related death. Secondary outcomes included overall survival and PRS distribution across genetic subgroups.

**Results:**

A total of 414 White women (mean [SD] age, 53.7 [9.7] years; 211 [51.0%] with postmenopausal status) with ILC were tested. No significant associations were found between germline variant subgroups and patients’ characteristics. PVs were identified in 46 patients (11.1%), with 20 (4.8%) carrying variants in moderate- to high-risk BC genes (*ATM, BARD1, BRCA1, BRCA2, CDH1, CHEK2, NF1, FANCM, PALB2, RAD51C, RAD51D, STK11, TP53, *and* PTEN*). The group of women carrying PVs in moderate- to high-risk BC genes had significantly reduced 5-year BC-free survival compared with the rest of cohort (62.2% [95% CI, 32.3%-82.0%] vs 92.1% [95% CI, 87.6%-95.0%]; hazard ratio, 3.91; 95% CI, 1.99-7.67; *P* < .001). PRS analysis did not reveal statistically significant differences in relapse risk across quartiles of PRS, and no association was found between PRSs and germline variant status.

**Conclusions and Relevance:**

This cohort study of women with primary ILC identified a clinically relevant subset of patients carrying moderate- to high-risk germline PVs who exhibited an increased risk of early relapse. Although PRSs did not show prognostic value in this setting, multigene panel testing findings may refine genetic counseling and inform surveillance and therapeutic strategies in lobular breast tumors.

## Key Points

**Question:**

What is the prevalence and clinical relevance of germline pathogenic and likely pathogenic variants and polygenic risk scores in women with invasive lobular carcinoma (ILC)?

**Findings:**

In this cohort study of 414 women with primary ILC, 4.8% carried germline pathogenic variants in moderate- and high-risk breast cancer predisposition genes, which were associated with a significantly higher rate of early breast cancer relapse. No significant associations were observed between polygenic risk scores and prognosis.

**Meaning:**

These findings suggest that multigene panel testing may identify a subset of patients with ILC who could benefit from intensified surveillance and targeted therapeutic strategies.

## Teaser

This cohort study evaluates the outcomes of germline pathogenic variants associated with increased breast cancer risk in the prognosis of women with primary invasive lobular carcinoma.

## Introduction

Invasive lobular carcinoma (ILC) represents the second most common histologic subtype of breast cancer (BC), accounting for approximately 10% to 15% of all cases.^1^ ILC is typically characterized by a diffuse growth pattern, reduced cell cohesion, and frequent hormone receptor positivity, but it often lacks *ERBB2 *(previously* HER2/neu*) (OMIM 164870) amplification and shows a lower proliferative index. These characteristics contribute to challenges in early detection through standard imaging and to differences in response to systemic therapies compared with invasive ductal carcinoma (IDC).^1^ Established risk factors for ILC include hormone replacement therapy, alcohol consumption, early menarche (before 12 years of age), late-onset menopause, nulliparity or low parity, delayed age at first childbirth (>30 years), and a positive family history of BC.^2^

Germline pathogenic or likely pathogenic variants (PVs) in several genes, most notably *CDH1* (OMIM 192090), are strongly associated with increased ILC risk. A case-control study^3^ reported a notable prevalence of germline PVs in *BRCA2 *(OMIM 600185)*, CDH1, CHEK2 *(OMIM 604373), and *PALB2* (OMIM 610355) among individuals with ILC, particularly in women diagnosed before the age of 40 years. These findings were subsequently corroborated by Yadav et al,^4^ who analyzed a larger ILC cohort and confirmed a similar distribution of PVs, reinforcing the role of these genes in lobular BC predisposition. In a more recent case-control analysis, Yadav et al^4^ identified *CDH1* and *BRCA2* germline PVs as conferring high risk for ILC, whereas *CHEK2*, *ATM *(OMIM 607585), and *PALB2* were associated with a moderate risk. Conversely, *BRCA1* (OMIM 114480) PVs were not significantly associated with ILC susceptibility. Additionally, *CDH1* PVs were significantly more frequent in ILC compared with IDC in contrast to *BRCA1*.

To our knowledge, this study aims for the first time to evaluate the outcomes of germline PVs associated with increased BC risk for the prognosis of patients performing multigene panel testing (MGPT) in a large cohort of women diagnosed with primary ILC. As a secondary objective, we evaluated the potential of polygenic risk scores (PRS) as prognostic markers in ILC. Improving genetic risk stratification in ILC could inform tailored surveillance strategies and contribute to more personalized risk-reduction interventions. Moreover, germline findings may have therapeutic relevance, particularly in the context of emerging targeted treatments (eg, PARP [poly(adenosine diphosphate-ribose) polymerase] inhibitors for *BRCA*-mutated tumors) and in refining eligibility for clinical trials.

## Methods

### Study Participants

This longitudinal, prospective cohort study analyzed 113 genes between May 16, 2022, and January 31, 2025, using blood samples collected from women with postoperative histopathologic diagnosis of ILC at the European Institute of Oncology. All patients with a confirmed diagnosis of primary ILC were considered eligible for screening. The collection of blood samples followed both prospective and retrospective procedures. For patients prospectively studied from May 2022 onward (219 women), blood samples were collected at enrollment and analyzed for germline variants. For patients studied retrospectively before study initiation (195 women), genetic testing was performed on blood samples obtained at the time of diagnosis and stored in the institutional biobank. Clinical, histopathologic, personal, and family history data were systematically recorded in a dedicated institutional database. Biological samples (whole blood) were stored in the institute’s biobank. Follow-up information was obtained through review of medical records, pathology reports, and structured telephone interviews conducted by trained personnel. The cutoff date for outcome ascertainment was January 31, 2023. All participants were offered both genetic and psychological counseling. The study was registered on ClinicalTrials.gov.^5^ This cohort study was approved by the Ethics Committee of the European Institute of Oncology, and written informed consent was obtained from all eligible participants. The study was conducted in accordance with the Strengthening the Reporting of Genetic Association Studies (STREGA) reporting guideline.

### DNA Extraction and Quantification

Genomic DNA was extracted from peripheral blood using a nucleic acid extractor (MagCore Super Automated Nucleic Acid Extractor, Diatech) following the manufacturer’s protocols. Initial quantification was performed with a nucleic acid purification system (MagCore, Diatech), whereas DNA concentration for next-generation sequencing library preparation was assessed using a DNA quantification assay (Qubit dsDNA HS Assay Kit, Life Technologies) and a fluorometer (Qubit 3.0 Fluorometer, Life Technologies).

### Library Preparation and Next-Generation Sequencing 

Samples were analyzed using a cancer solution panel (TruSight Hereditary Cancer Solution Panel, Illumina), targeting 113 genes associated with hereditary breast, ovarian, colorectal, and gastric cancers along with 125 single-nucleotide polymorphisms. Libraries were prepared using a sequencing solution (Illumina DNA Prep with Enrichment, Illumina) with 100 ng of genomic DNA (Qubit quantified). The panel covers coding regions and 20 base pairs (bp) of exon-flanking sequences. Libraries were quantified (4200 TapeStation, Agilent Technologies, and Qubit 3.0), diluted to 4 nM, denatured, and loaded (14 pM, 3% PhiX) onto a benchtop sequencer (Illumina MiSeq, Illumina) using a reagent kit (MiSeq V2 kit, Illumina) (2 × 150 cycles).

Data were analyzed with a genomic platform (SOPHiA DDM platform, SOPHiA Genetics) using the germline pipeline to detect SNVs, indels, and copy number variations. PRSs were also evaluated as described by Huntley et al^6^ and Padrik et al,^7^ using a single-nucleotide polymorphism array (Axoim, Thermo Fisher Scientific) interrogating more than 850 000 markers.

### Variant Classification

Variants were classified as pathogenic, likely pathogenic, variant of uncertain significance (VUS), likely benign, or benign according to International Agency for Research on Cancer guidelines and American College of Medical Genetics and Genomics and the Association for Molecular Pathology recommendations.^8,9^ Interpretation was supported by databases and in silico tools, including ClinVar, *BRCA* Exchange, LOVD, InSiGHT, and Varsome.

### Main Analysis

Patients were classified into 2 groups based on germline variant status and their established association with BC risk: (1) carriers of moderate- to high-risk BC variants and (2) noncarriers of moderate- to high-risk BC variants. The moderate- to high-risk group included women carrying at least 1 PV in genes known to confer increased BC risk (*ATM, BARD1 *[OMIM 601593]*, BRCA1, BRCA2, CDH1, CHEK2, NF1 *[OMIM 613113]*, FANCM *[OMIM 609644]*, PALB2, RAD51C *[OMIM 602774]*, RAD51D *[OMIM 602954]*, STK11 *[OMIM 602216]*, TP53 *[OMIM 191170], and *PTEN* [OMIM 601728]). The noncarriers of moderate- to high-risk BC variants group included patients carrying PVs in genes not currently associated with increased BC risk, those with 1 or more VUSs in any of the analyzed genes, and those with no detected variants. In addition to this classification, we further categorized carriers of moderate- to high-risk BC variants into 2 groups: carriers of variants in high-penetrance genes (*BRCA1, BRCA2, *and* CDH1*) and carriers of variants in medium-penetrance genes (*ATM, CHEK2, PALB2, *and* NF1*).

Patient and tumor characteristics were summarized using absolute and relative frequencies and compared using the χ^2^ test. PRSs were described as means with 95% CIs, and between-group differences were assessed using 2-sided independent samples *t* tests.

The primary outcome was BC-free survival (BCFS), defined as the time from surgery to the first occurrence of any of the following events: invasive recurrence in the ipsilateral breast, locoregional recurrence, distant recurrence, ductal carcinoma in situ in either breast, or death due to BC. Patients without events were censored at the date of last follow-up. BCFS was chosen as the primary end point because the study primarily aimed to evaluate the outcome of germline PVs associated with increased BC risk and the prognosis of patients with ILC.

BCFS curves for each group (carriers of moderate- to high-risk BC variants vs noncarriers of moderate- to high-risk BC variants) were estimated using the Kaplan-Meier method. Hazard ratios (HRs) with 95% CIs comparing the 2 groups were calculated using univariable Cox proportional hazards regression models, with risk group as the main exposure. Tumor size, nodal status, and histologic grade were not included as adjustment variables because they were considered potential mediators on the causal pathway between germline variants and clinical outcomes rather than confounders. Adjusting for these factors could therefore lead to overadjustment and attenuation of the overall effect of germline variants. Analyses were conducted without adjustment for tumor-related characteristics to estimate the total effect of germline PVs on recurrence and overall survival. The secondary outcome was overall survival, defined as the time from surgery to death from any cause.

### PRS Analysis

For PRS analysis, patients were categorized into quartiles (quartiles 1-4) based on the distribution of PRSs. The association between each PRS and BCFS was evaluated using a Cox proportional hazards regression model, with the PRS quartile group included as a categorical covariate and quartile 1 serving as the reference category. A test for linear trend across quartiles was also conducted by modeling the quartile group as an ordinal numeric variable within the Cox proportional hazards regression model, and the *P* value associated with the covariate parameter estimate was extracted. This analysis was repeated for each individual PRS.

### Statistical Analysis

All statistical tests were 2-sided, and *P *< .05 was considered statistically significant. Statistical analyses were performed in January 2026 using SAS software, version 9.4 (SAS Institute Inc). We selected from the PGSCATALOG repository the well-known PRS 313 together with the other available PRSs computed on patients with estrogen receptor–positive BC (because most ILC cases are estrogen receptor positive). The selected PRSs were previously described.^6,7^

## Results

### Germline Variants in ILC

**Figure 1.**
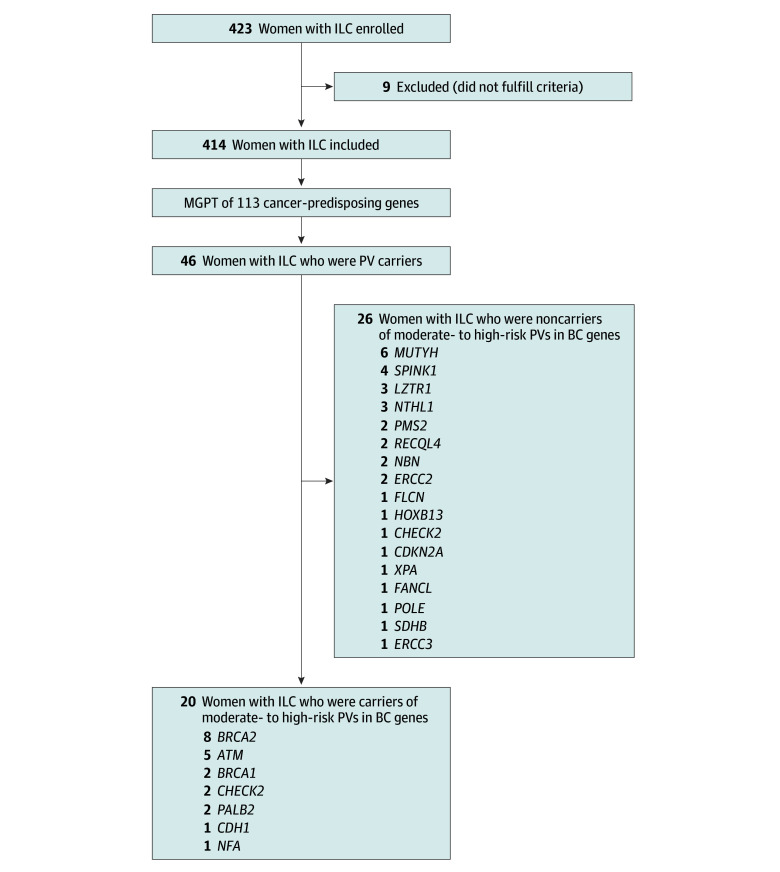
Flow Diagram of Patient Enrollment and Germline Testing Results This flow diagram shows patient enrollment and germline genetic testing results in women with invasive lobular carcinoma (ILC). Of 423 patients initially enrolled, 414 met eligibility criteria and were included in the analysis, whereas 9 patients were excluded. Multigene panel testing (MGPT) of 113 cancer predisposition genes identified germline pathogenic variants (PVs) in 46 patients (11.1%). Among them, 20 patients (4.8%) carried variants in moderate- or high-risk breast cancer (BC) predisposition genes (*BRCA2, ATM, BRCA1, CHEK2, PALB2, CDH1, *and* NF1*), whereas 26 patients carried variants in other cancer susceptibility genes.

From an initial consecutive cohort of 423 White women with primary ILC, 414 patients were included in the genetic screening analysis. Nine patients were excluded due to failure to meet clinical eligibility criteria. The overall study design, patient selection, and distribution of genetic findings are summarized in Figure 1. Among the 414 included patients (mean [SD] age, 53.7 [9.7] years; 211 [51.0%] with postmenopausal status), 12 (2.9%) had undergone breast surgery for a second primary BC (11 of the noncarriers of moderate- to high-risk BC variants group and 1 of the carriers of moderate- to high-risk BC variants group), whereas the remaining women underwent surgery for a first primary tumor. Regarding germline findings, 38 patients (9.2%) carried at least 1 PV and at least 1 VUS, 8 patients (1.9%) carried only PVs, 340 (82.1%) carried at least 1 VUS without PVs, and 28 (6.8%) had no detectable variants. Overall, PVs were identified in 46 of 414 patients, corresponding to a prevalence of 11.1%. Among these PVs, PVs in genes classified as moderate to high risk for BC were detected in 20 patients (4.8%).

Among genes known to be associated with increased BC risk, PVs were identified in *BRCA2* in 8 women (1.9%); *ATM* in 5 women (1.2%); *BRCA1*, *CHEK2*, and *PALB2* in 2 women each (0.5%); and *CDH1* and *NF1* in 1 woman each (0.2%). One patient harbored PVs in 2 different moderate- to high-risk genes (*ATM* and *CHEK2*). A detailed list of identified PVs is provided in eTable 1 in Supplement 1.

Among genes not currently recognized as associated with increased BC risk, PVs were found in *MUTYH* (OMIM 604933) in 6 women (1.4%) (notably, 1 patient carried a homozygous variant); *SPINK1 *(OMIM 167790), *LZTR1 *(OMIM 600574), and *NTHL1* (OMIM 602656) in 3 women each (0.7%); *PMS2 *(OMIM 600259), *RECQL4 *(OMIM 603780), *NBN *(OMIM 602667), and *ERCC2* (OMIM 126340) in 2 women each (0.5%); and *FLCN *(OMIM 607273), *HOXB13 *(OMIM 604607), *CDKN2A *(OMIM 600160), *FANCE *(OMIM 613976), *CTRC *(OMIM 601405), *XPA *(OMIM 611153), *FANCL *(OMIM 608111, *POLE *(OMIM 174762), *SDHB *(OMIM 185470)*, *and* ERCC3 *(OMIM 133510) in 1 woman each (0.2%). In total, these variants were identified in 26 patients, including 3 individuals who carried PVs in 2 different genes (Figure 1). No significant associations were found between germline variant subgroups and the clinicopathologic characteristics, family history, surgical procedures, or adjuvant therapies among patients with ILC (Table 1).

**Table 1.** Patient Characteristics

Characteristic	No. (%) of patients^a^			*P* value
	Overall (N = 414)	Carriers of moderate- to high-risk BC genes (n = 20)	Noncarriers of moderate- to high-risk BC genes (n = 394)	
Year of surgery				
Before 2022	167 (40.3)	12 (60.0)	155 (39.3)	.07
2022 or after	247 (59.7)	8 (40.0)	239 (60.7)	
Age at surgery, y				
<35	1 (0.2)	0	1 (0.3)	.89
35-50	198 (47.8)	10 (50.0)	188 (47.7)	
51-65	152 (36.7)	6 (30.0)	146 (37.1)	
>65	63 (15.2)	4 (20.0)	59 (15.0)	
Age at surgery, mean (SD), y	53.77 (9.71)	54.3 (11.33)	53.74 (9.64)	.80
Menopausal status				
Premenopausal	198 (47.8)	5 (25.0)	193 (49.0)	.07
Postmenopausal	211 (51)	13 (65.0)	198 (50.3)	
Unknown	5 (1.2)	2 (10.0)	3 (0.8)	
Familiarity for breast cancer				
No	225 (54.3)	11 (55.0)	214 (54.3)	.96
Yes	188 (45.4)	9 (45.0)	179 (45.4)	
Unknown	1 (0.2)	0	1 (0.3)	
Familiarity for ovary cancer				
No	373 (90.1)	18 (90.0)	355 (90.1)	.92
Yes	23 (5.6)	1 (5.0)	22 (5.6)	
Unknown	18 (4.3)	1 (5.0)	17 (4.3)	
Previous cancer				
No	356 (86)	15 (75.0)	341 (86.5)	.07
Yes	49 (11.8)	5 (25.0)	44 (11.2)	
Unknown	9 (2.2)	0	9 (2.3)	
Neoadjuvant CT				
No	382 (92.3)	17 (85.0)	365 (92.6)	.14
Yes	28 (6.8)	3 (15.0)	25 (6.3)	
Unknown	4 (1)	0	4 (1)	
Type of surgery				
Mastectomy	113 (27.3)	8 (40.0)	105 (26.6)	.51
Nipple-sparing mastectomy	149 (36)	5 (25.0)	144 (36.5)	
Quadrantectomy	144 (34.8)	7 (35.0)	137 (34.8)	
Other	7 (1.7)	0	7 (1.8)	
Unknown	1 (0.2)	0	1 (0.3)	
Tumor size, cm				
<2	192 (46.4)	11 (55.0)	181 (45.9)	.42
≥2	201 (48.6)	8 (40.0)	193 (49.0)	
Unknown	21 (5.1)	1 (5.0)	20 (5.1)	
Side of surgery				
Unilateral	401 (96.9)	20 (100)	381 (96.7)	.47
Bilateral	10 (2.4)	0	10 (2.5)	
Unknown	3 (0.7)	0	3 (0.8)	
Sentinel lymph node biopsy				
No	61 (14.7)	1 (5.0)	60 (15.2)	.23
Yes	349 (84.3)	18 (90.0)	331 (84.0)	
Unknown	4 (1)	1 (5.0)	3 (0.8)	
pT				
pTx/is	11 (2.7)	1 (5.0)	10 (2.5)	.35
pT0	1 (0.2)	0	1 (0.3)	
pT1	206 (49.8)	11 (55.0)	195 (49.5)	
pT2	151 (36.5)	7 (35.0)	144 (36.5)	
pT3/4	45 (10.9)	1 (5.0)	44 (11.2)	
pN				
pNx	19 (4.6)	NA	19 (4.8)	.35
pN0	244 (58.9)	15 (75.0)	229 (58.1)	
pN1	90 (21.7)	2 (10.0)	88 (22.3)	
pN2/3	61 (14.7)	3 (15.0)	58 (14.7)	
Stage				
Early breast cancer	317 (76.6)	16 (80.0)	301 (76.4)	.79
Locally advanced breast cancer	70 (16.9)	3 (15.0)	67 (17.0)	
Unknown	27 (6.5)	1 (5.0)	26 (6.6)	
Grade				
1	55 (13.3)	3 (15.0)	52 (13.2)	.92
2	277 (66.9)	12 (60.0)	265 (67.3)	
3	38 (9.2)	2 (10.0)	36 (9.1)	
Unknown	44 (10.6)	3 (15.0)	41 (10.4)	
Subtype				
Luminal A	308 (74.4)	18 (90.0)	290 (73.6)	.45
Luminal B (Ki-67 ≥20%)	73 (17.6)	1 (5.0)	72 (18.3)	
Luminal B (*ERBB2* positive)	17 (4.1)	1 (5.0)	16 (4.1)	
Triple negative	2 (0.5)	0	2 (0.5)	
Unknown	14 (3.4)	0	14 (3.6)	
ER or PR, %				.07
Not expressed (both 0)	2 (0.5)	0	2 (0.5)	
Incompletely expressed (ER <50% or PR <50%)	128 (30.9)	11 (55.0)	117 (29.7)	
Highly expressed (ER ≥50% and PR ≥50%)	272 (65.7)	9 (45.0)	263 (66.8)	
Unknown	12 (2.9)	0	12 (3.0)	
Ki-67, %				
<20	321 (77.5)	19 (95.0)	302 (76.6)	.08
≥20	82 (19.8)	1 (5.0)	81 (20.6)	
Unknown	11 (2.7)	0	11 (2.8)	
*ERBB2* status				
Negative (0)	240 (58)	12 (60.0)	228 (57.9)	.98
Low (1+ or 2+ with negative or unknown FISH)	146 (35.3)	7 (35.0)	139 (35.3)	
Positive (2+ with positive FISH or 3+)	17 (4.1)	1 (5.0)	16 (4.1)	
Unknown	11 (2.7)	0	11 (2.8)	
Vascular invasion				
No	353 (85.3)	18 (90.0)	335 (85)	.42
Yes	12 (2.9)	0	12 (3.0)	
Unknown	49 (11.8)	2 (10.0)	47 (11.9)	
Systemic therapy				
No therapy	7 (1.7)	0	7 (1.8)	.82
CT	4 (1)	0	4 (1.0)	
ET	234 (56.5)	10 (50.0)	224 (56.9)	
CT and ET	66 (15.9)	4 (20.0)	62 (15.7)	
Unknown	103 (24.9)	6 (30.0)	97 (24.6)	
Second primary tumors				
No event	396 (95.7)	19 (95.0)	377 (95.7)	.88
Event	18 (4.3)	1 (5.0)	17 (4.3)	

Abbreviations: CT, chemotherapy; ER, estrogen receptor; ET, endocrine therapy; FISH, fluorescence in situ hybridization; NA, not applicable; PR, progesterone receptor.

^a^ Unless otherwise indicated.

### Clinical Follow-Up

This study aimed to evaluate early BC relapse, with a median (IQR) follow-up of 3.39 (2.61-6.53) years. Both overall survival and BCFS were assessed; however, overall survival could not be reliably estimated due to the relatively short duration of follow-up: only 4 events were observed in the noncarriers of moderate- to high-risk risk variants group.

**Figure 2.**
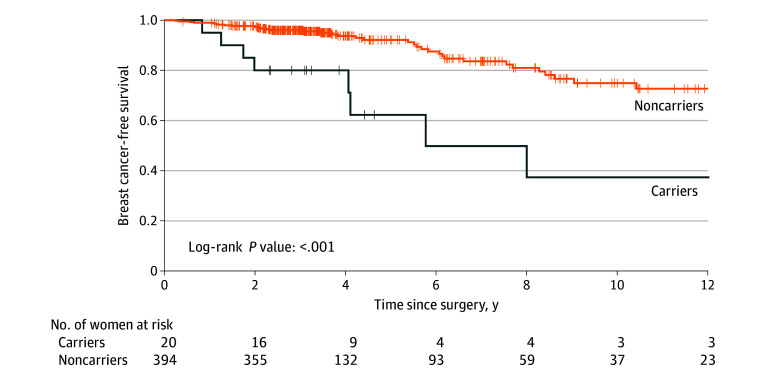
Kaplan-Meier Curve of Breast Cancer–Free Survival According to Germline Variant Status Kaplan-Meier estimates of breast cancer–free survival in patients with invasive lobular carcinoma stratified by germline variant status. Carriers of pathogenic variants in moderate- or high-risk breast cancer predisposition genes are compared with noncarriers. Time was calculated from surgery to ipsilateral recurrence, contralateral breast cancer, distant metastasis, or breast cancer–related death. Tick marks indicate censored observations. *P* values were calculated using the log-rank test.

With respect to BCFS, 11 events (55%) were observed in the moderate- to high-risk group (55.0%) and 47 events (11.9%) in noncarriers of moderate- to high-risk risk variants group. Similar trends were observed when considering 10-year BCFS estimates (Table 2). Kaplan-Meier analysis demonstrated a significant association between moderate- to high-risk germline status and BC relapse, including locoregional and/or distant recurrence (median recurrence-free survival in carriers: 5.77 [95% CI, 4.07-12.75] years; median recurrence-free survival in noncarriers: 14.54 [95% CI, 9.05-16.81] years; log-rank *P* < .001) (Figure 2). The Cox proportional hazards regression model yielded an HR of 3.91 (95% CI, 1.99-7.67), meaning that the BC-related recurrence rate in the moderate- to high-risk group was significantly higher than in the noncarriers of moderate- to high-risk BC variants group.

**Table 2.** BCFS by Group

Outcome	Carriers of moderate- to high-risk BC genes (n = 20)	Noncarriers of moderate- to high-risk BC genes (n = 394)
Observed events, No. (%)		
Local recurrence	5 (25.0)	19 (4.8)
Regional recurrence	1 (5.0)	1 (0.2)
Distant metastases	3 (15.0)	18 (4.6)
Other events	2 (10.0)	9 (2.3)
Total	11 (55.0)	(47 (11.9)
BCFS, median (95% CI)		
5-y	62.2 (32.3-82.0)	92.1 (87.6-95.0)
10-y	37.3 (10.2-65.3)	74.9 (64.9-82.4)
Crude HR (95% CI)^a^	3.9 (2.0-7.7)	1.00 [Reference]

Abbreviations: BC, breast cancer; BCFS, breast cancer–free survival; HR, hazard ratio.

^a^ The HR and 95% CI were obtained from a Cox proportional hazards regression model with group as covariate.

As exploratory analyses, stratified Kaplan-Meier analyses with log-rank tests were performed according to local stage and gene penetrance. Given the limited statistical power related to the small sample size and the increased risk of false-positive findings inherent to subgroup analyses, results should be interpreted with caution. However, considering the exploratory nature of these analyses, no correction for multiple comparisons was applied.

When stratified by local stage, recurrence-free survival differed significantly between carriers and noncarriers in early BC (median recurrence-free survival in carriers: 5.77 [95% CI, 4.07-12.75] years; median recurrence-free survival in noncarriers: 13.93 [95% CI, 13.30-15.95] years; log-rank *P* < .001) (eFigure 1A in Supplement 1) but not in locally advanced BC (eFigure 1B in Supplement 1). When stratified by gene penetrance, no significant differences were observed between moderate- and low-penetrance groups (eFigure 2A in Supplement 1) or between high- and moderate-penetrance groups (eFigure 2C in Supplement 1), whereas recurrence-free survival significantly differed between high- and low-penetrance groups (median recurrence-free survival in high-penetrance group: 4.11 [95% CI, 1.74-14.51] years; median recurrence-free survival in high-penetrance group: 14.54 [95% CI, 9.05-16.81] years; log-rank *P* < .001) (eFigure 2B in Supplement 1).

### PRS and Prognosis

**Figure 3.**
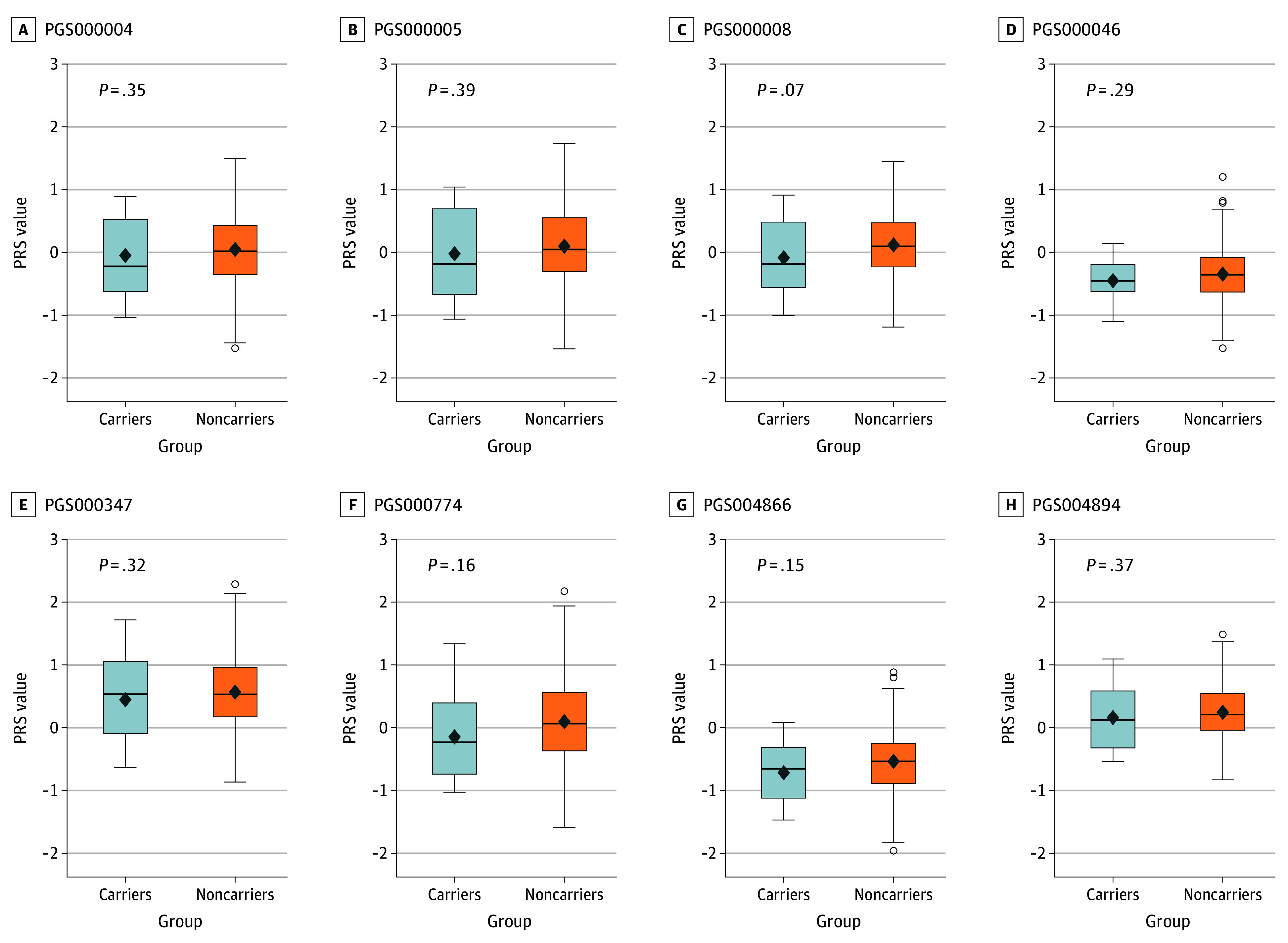
Box Plots of Polygenic Risk Score (PRS) Distribution by Germline Variant Status Distribution of PRS in patients with invasive lobular carcinoma according to germline pathogenic variant status (carriers vs noncarriers). Eight published polygenic score models were evaluated (PGS000004, PGS000005, PGS000008, PGS000046, PGS000347, PGS000774, PGS004866, and PGS004894). Boxes represent the IQR, center lines indicate medians, and whiskers indicate ranges excluding outliers. Diamonds indicate mean values and circles indicate the outliers. *P* values correspond to comparisons between groups.

Figure 3 displays box plots of PRS distributions across the 2 genetic risk groups for each PRS, along with *P* values derived from independent samples *t*-tests comparing mean PRSs between groups. No statistically significant differences were observed for any PRS.

eTable 2 in Supplement 1 reports the HRs and 95% CIs for each comparison between PRS quartiles (quartiles 2-4) vs the reference group (quartile 1), including *P* values for both individual quartile comparisons and linear trends across quartiles. Overall, none of the 8 PRSs demonstrated a statistically significant linear trend across quartiles.

## Discussion

### Frequency of Germline PVs

To our knowledge, this is the largest single-center, longitudinal cohort study to report results from MGPT, including 113 genes in women with ILC. Focusing on genes associated with moderate to high BC risk, approximately 5% of women with primary ILC in our cohort carried germline variants conferring a substantially increased predisposition to BC.

Comparable frequencies have been reported in previous studies.^3,10^ A previous study analyzing moderate- and high-risk genes (*BRCA1/2, CHEK2, PALB2, CDH1,* and *TP53*) in 1434 women with ILC identified PVs in 4.4% of cases.^3^ Another study, using a multigene panel of 33 genes within the BRIDGES study, tested 302 ILC cases and identified 35 germline PVs (11.6%), of which 7.4% were classified as clinically actionable.^10^ However, detailed clinicopathologic characteristics and BC relapse outcomes were not reported in that study. Similarly, up to 49 genes in 3437 women with ILC have been evaluated by Yadav and colleagues,^4^ who reported a cumulative frequency of 6.5% for PVs in known BC predisposition genes; data on the overall frequency of all detected PVs were not available.

In our cohort, the most frequently involved genes were *BRCA2* (1.9%) and *ATM* (1.2%). These findings are consistent with prior reports: Yadav et al^4^ observed PVs in *BRCA2* (2.1%), *CHEK2* (1.2%), and *ATM* (1.0%); van Veen et al^10^ reported variants in *BRCA2* (5.6%), *BRCA1* (1.6%), and *ATM* (1.3%); and Petridis et al^3^ identified variants in *BRCA2* (1.8%) and *CHEK2* (1.3%). Collectively, these results support a strong genetic component in ILC susceptibility, primarily driven by PVs in *BRCA2, CHEK2, ATM,* and *CDH1*. PVs in other genes, such as *BRCA1, PALB2,* and *TP53*, may also contribute to ILC risk, although their association appears to be stronger with IDC; nevertheless, their relevance in ILC should not be overlooked.

The role of low-risk genes in predisposing women with ILC to BC remains less well defined. Individually, these genes are thought to confer only modest increases in BC risk, and additional studies are needed to clarify whether combinations of low-risk variants or interactions with additional genetic and environmental factors may more accurately define BC predisposition in this population.

### BC-Free Survival

In our study, more than half (55.0%) of women with ILC who carried moderate- to high-risk germline PVs experienced BC relapse during follow-up. In IDC, germline PVs, particularly in the *BRCA1* gene, are known to significantly affect prognosis. These variants are often associated with more aggressive tumor phenotypes, such as triple-negative BC (TNBC), increased risk of recurrence, and greater likelihood of lymph node involvement. However, they also confer sensitivity to targeted therapies, including PARP inhibitors, which may improve survival in selected settings (eg, early-stage TNBC or metastatic disease).^11,12^

To date, to our knowledge, no studies have specifically evaluated the prognostic impact of moderate- to high-risk germline ILC PVs. Our findings suggest that ILC patients carrying such variants may be at increased risk of early BC relapse. This observation is particularly noteworthy given that most ILCs are of luminal subtype and typically follow a more indolent course compared with TNBC.

However, our data indicate that a subset of lobular tumors associated with germline predisposition genes recurred early (within 3 years), mirroring the relapse dynamics seen in more aggressive subtypes, such as TNBC. This subgroup warrants closer clinical attention in terms of both follow-up strategies and the development of a more refined molecular classification to guide personalized management.

### PRS as Prognostic Marker

We did not observe any significant association between PRSs and clinical outcomes in women with ILC. In our cohort, none of the 8 PRSs analyzed showed statistically significant differences between carriers of moderate- to high-risk BC variants group and noncarriers of moderate- to high-risk BC variants groups. We also did not find BCFS differences across PRS quartiles. These findings are consistent with those reported by Xin et al,^11^ who analyzed more than 26 000 patients with cancer across the UK Biobank and The Cancer Genome Atlas cohorts. Despite confirming that PRSs are useful in stratifying cancer risk, Xin et al^11^ found no association between site-specific PRSs and cancer-specific survival in any tumor type, including BC.^13^

Our data reinforce this conclusion within the specific setting of ILC: germline mutations in moderate- to high-risk genes (eg, *BRCA2*, *ATM*, and *CHEK2*) did not correlate with PRS values, and PRS-based stratification failed to identify patients with worse prognosis. These results highlight the current limitation of PRSs as prognostic tools and suggest that novel, outcome-oriented polygenic models may be needed for personalized prognostication in ILC.

### Limitations

This study presents several limitations that should be acknowledged. First, despite being one of the largest single-center cohorts of patients with ILC undergoing MGPT with 113 genes, the absolute number of patients carrying moderate- to high-risk PVs remains relatively small, limiting the power of subgroup analyses and generalizability of prognostic findings. Second, although follow-up was prospectively collected, the median follow-up time of 3.39 years may be insufficient to fully capture late recurrences, which are not uncommon in hormone receptor–positive ILC. Furthermore, we will update the follow-up until 10 years. Third, the study was conducted in a single institution, potentially introducing referral bias and limiting external validity. Fourth, although PRS analyses followed standardized methods, the polygenic models applied were not specifically developed for ILC and may not adequately reflect risk or prognosis in this histologic subtype. Fifth, the lack of functional validation for many of the identified variants, particularly in genes not traditionally associated with BC risk, limits the interpretation of their clinical relevance.

## Conclusions

This cohort study provides detailed insight into the landscape of MGPT in women with ILC. Our findings suggest the existence of a distinct subset of ILC characterized by germline PVs in BC predisposition genes, which may be associated with an increased risk of early locoregional or distant relapse. These results, if further validated in larger cohorts allowing for effect comparison between PVs carriers at high and moderate BC risk, will have potential implications for genetic counseling by refining individual risk stratification, ultimately supporting clinicians in tailoring surveillance strategies and considering emerging targeted adjuvant therapies for genetically defined high-risk patients with ILC.

## Notes

Supplement. **eTable 1.** List of the identified germline pathogenic/likely pathogenic variants in the screened women **eTable 2.** Hazard ratios (HR) with 95% confidence intervals (CI) for breast cancer-invasive disease-free survival (iDFS) obtained from Cox proportional hazard models fitted for each polygenic risk score (PRS) **eFigure 1.** Breast Cancer–Free Survival According to Germline Variant Status Stratified by Local Stage **eFigure 2.** Breast Cancer–Free Survival According to Penetrance

Supplement 2.
Data Sharing Statement
