# Supplementary material for: Germline Multigene Panel Testing in Women With Invasive Lobular Cancer
Source: JAMA Netw Open. 2026 Jul 8;9(7):e2621705. doi: 10.1001/jamanetworkopen.2026.21705 (PMC13347242; doi:10.1001/jamanetworkopen.2026.21705)
Supplement: Supplement. — eTable 1. List of the identified germline pathogenic/likely pathogenic variants in the screened women eTable 2. Hazard ratios (HR) with 95% confidence intervals (CI) for breast cancer-invasive disease-free survival (iDFS) obtained from Cox proportional hazard models fitted for each polygenic risk score (PRS) eFigure 1. Breast Cancer–Free Survival According to Germline Variant Status Stratified by Local Stage eFigure 2. Breast Cancer–Free Survival According to Penetrance [file jamanetwopen-e2621705-s001.pdf]

Supplemental Online Content

Corso G, Marino E, Fava F, et al. Germline multigene panel testing in women with invasive lobular cancer. *JAMA Netw Open*. 2026;9(7):e2621705.
doi:10.1001/jamanetworkopen.2026.21705

eTable 1. List of the identified germline pathogenic/likely pathogenic variants in the screened women

eTable 2. Hazard ratios (HR) with 95% confidence intervals (CI) for breast cancer-invasive disease-free survival (iDFS) obtained from Cox proportional hazard models fitted for each polygenic risk score (PRS)

eFigure 1. Breast Cancer–Free Survival According to Germline Variant Status Stratified by Local Stage

eFigure 2. Breast Cancer–Free Survival According to Penetrance

This supplemental material has been provided by the authors to give readers additional information about their work.

© 2026 Corson G et al. *JAMA Network Open*.

eTable 1. List of the identified germline pathogenic/likely pathogenic variants in the screened women

Code	Gene	Nomenclature
1730-008	*SPINK1*	c.101A>G (p.Asn34Ser)
1730-009	*BRCA1*	c.1088del p.(Asn363Ilefs*11)
1730-028	*BRCA2*	c.7857G>A (p.Trp2619Ter)
1730-036	*MUTYH*	c.1187G>A (p.Gly396Asp)
1730-043	*PMS2*	c.1687C>T p.(Arg563Ter)
1730-051	*FLCN*	c.1285dup (p.His429ProfsTer27)
1730-051	*MUTYH*	c.734G>A (p.Arg245His)
1730-057	*HOXB13*	c.251G>A p.(Gly84Glu)
1730-069	*CDH1*	c.2042T>G p.(Leu681Ter)
1730-093	*BRCA1*	c.514del (p.Gln172AsnfsTer62)
1730-097	*ATM*	c.6658C>T(p.Gln2220Ter)
1730-102	*CDKN2A*	c.301G>Tp.(Gly101Trp)
1730-108	*BRCA2*	c.6468_6469del p.(Gln2157IlefsTer18)
1730-108	*LZTR1*	c.1397G>A p.(Arg466Gln)
1730-115	*RECQL4*	c.1887_1890del (p.Glu630AlafsTer59)
1730-116	*ATM*	c.6650_6657del p.(Phe2217SerfsTer29)
1730-124	*FANCE*	c.2T>C (p.Met1Thr)
1730-133	*CTRC*	c.217G>A p.(Ala73Thr)
1730-134	*CHEK2*	c.1100del (p.Thr367MetfsTer15)
1730-153	*NTHL1*	c.244C>T (p.Gln82Ter)
1730-156	*SPINK1*	c.101A>G (p.Asn34Ser)
1730-161	*NBN*	c.1142del (p.Pro381GlnfsTer23)
1730-168	*BRCA2*	c.3860del p.(Asn1287IlefsTer6)
1730-192	*RECQL4*	c.319C>T p.(Gln107Ter)
1730-202	*ATM*	c.8676dup(p.Ala2893CysfsTer3)
1730-208	*BRCA2*	c.7180A>T(p.Arg2394Ter)
1730-208	*ERCC2*	c.1973G>A(p.Arg658His)
1730-208	*XPA*	c.619C>T (p.Arg207Ter)
1730-213	*PALB2*	del exons 2-4
1730-218	*FANCL*	c.1022_1024del p.(Ile341_Cys342delinsSer)
1730-221	*ERCC2*	c.2164C>T p.(Arg722Trp)
1730-232	*PMS2*	c.137G>T p.(Ser46Ile)
1730-259	*NF1*	c.5248A>G p.(Lys1750Glu)
1730-270	*ERCC3*	c.1421dup p.(Asp474GlufsTer2)
1730-276	*POLE*	c.2599G>A(p.Val867Ile)
1730-288	*BRCA2*	c.9409_9412del(p.Thr3137TyrfsTer25)
1730-288	*NTHL1*	NTHL1: c.244C>T (p.Gln82Ter)
1730-319	*PALB2*	c.2559C>T p.(Gly853=)
1730-325	*NTHL1*	c.268C>T p.(Gln90Ter)

© 2026 Corson G et al. *JAMA Network Open*.

1730-328	*SDHB*	c.650G>A p.(Arg217His)
1730-335	*LZTR1*	c.27del p.(Gln10ArgfsTer15)
1730-335	*MUTYH*	c.1187G>A (p.Gly396Asp)
1730-342	*LZTR1*	c.1149+1G>T p.?
1730-346	*MUTYH**	c.1187G>A (p.Gly396Asp)
1730-349	*BRCA2*	c.5217_5220del p.(Tyr1739*)
1730-355	*ATM*	c.4906C>T p.(Gln1636Ter)
1730-367	*SPINK1*	c.101A>G (p.Asn34Ser)
1730-369	*NBN*	c.657_661del p.(Lys219AsnfsTer16)
1730-373	*SPINK1*	c.101A>G (p.Asn34Ser)
1730-378	*ATM*	c.8147T>C p.(Val2716Ala)
1730-378	*CHEK2*	c.470T>C p.(Ile157Thr)
1730-387	*MUTYH*	c.1038G>A p.(Ser346=)
1730-393	*BRCA2*	c.5722_5723del p.(Leu1908ArgfsTer2)
1730-402	*BRCA2*	c.7180A>T (p.Arg2394Ter)
1730-413	*MUTYH*	c.1187G>A p.(Gly396Asp)

*Homozygous germline variant

© 2026 Corson G et al. *JAMA Network Open*.

eTable 2. Hazard ratios (HR) with 95% confidence intervals (CI) for breast cancer-invasive disease-free survival (iDFS) obtained from Cox proportional hazard models fitted for each polygenic risk score (PRS)

Patients were categorized into four groups (Q1, Q2, Q3, Q4) according to the quartiles of the distribution of PRS values. The variable defining the quartile group was included as a covariate in the Cox model, with Q1 as the reference group. P-values for each HR and for linear trend across quartiles are provided.

PRS	Comparison	HR (95% CI)	P-value for HR	P-value for trend
PGS000004	Q2 vs. Q1	0.76 (0.35 to 1.65)	.49	.77
PGS000004	Q3 vs. Q1	0.77 (0.33 to 1.77)	.54	
PGS000004	Q4 vs. Q1	1.03 (0.50 to 2.13)	.93	
PGS000005	Q2 vs. Q1	0.76 (0.36 to 1.62)	.48	.92
PGS000005	Q3 vs. Q1	0.57 (0.25 to 1.31)	.19	
PGS000005	Q4 vs. Q1	0.97 (0.49 to 1.94)	.94	
PGS000008	Q2 vs. Q1	0.49 (0.23 to 1.07)	.07	.25
PGS000008	Q3 vs. Q1	0.57 (0.28 to 1.18)	.13	
PGS000008	Q4 vs. Q1	0.62 (0.31 to 1.24)	.17	
PGS000046	Q2 vs. Q1	1.51 (0.69 to 3.30)	.30	.50
PGS000046	Q3 vs. Q1	1.04 (0.46 to 2.36)	.93	
PGS000046	Q4 vs. Q1	1.48 (0.69 to 3.18)	.31	
PGS000347	Q2 vs. Q1	0.72 (0.34 to 1.53)	.40	.83
PGS000347	Q3 vs. Q1	0.51 (0.23 to 1.13)	.10	
PGS000347	Q4 vs. Q1	0.99 (0.48 to 2.01)	.97	
PGS000774	Q2 vs. Q1	0.89 (0.45 to 1.80)	.75	.07
PGS000774	Q3 vs. Q1	0.61 (0.29 to 1.31)	.21	
PGS000774	Q4 vs. Q1	0.54 (0.25 to 1.15)	.11	
PGS004866	Q2 vs. Q1	0.54 (0.25 to 1.15)	.11	0.31
PGS004866	Q3 vs. Q1	0.63 (0.31 to 1.27)	.19	

© 2026 Corson G et al. *JAMA Network Open*.

PRS	Comparison	HR (95% CI)	P-value for HR	P-value for trend
PGS004866	Q4 vs. Q1	0.64 (0.31 to 1.33)	.23	
PGS004894	Q2 vs. Q1	0.85 (0.40 to 1.81)	.67	.92
PGS004894	Q3 vs. Q1	0.55 (0.25 to 1.21)	.14	
PGS004894	Q4 vs. Q1	1.05 (0.53 to 2.08)	.89	

© 2026 Corson G et al. *JAMA Network Open*.

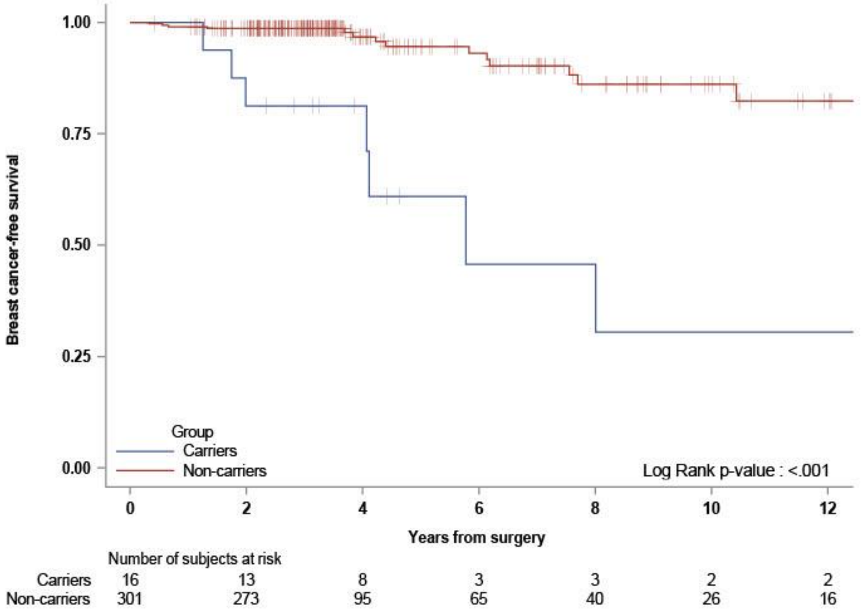

eFigure 1.

A

© 2026 Corson G et al. *JAMA Network Open*.

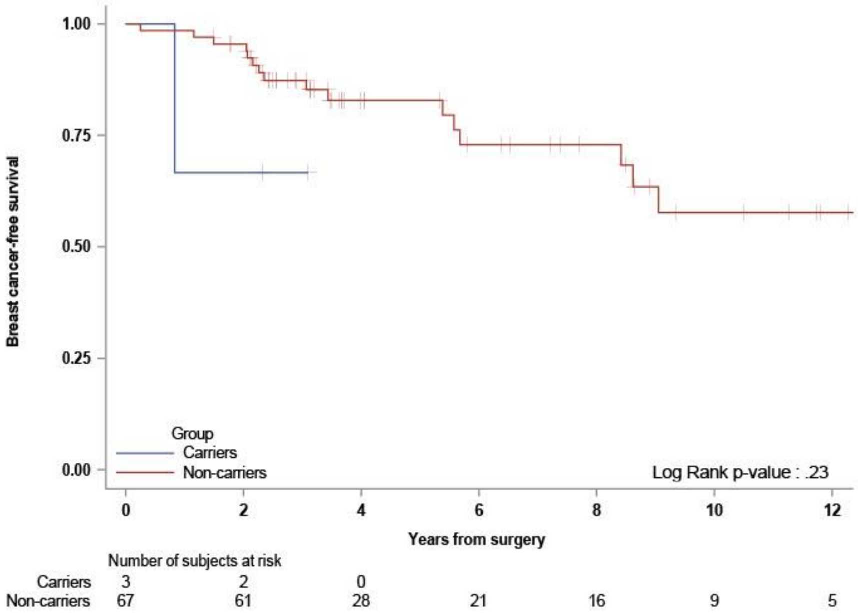

B.

© 2026 Corson G et al. *JAMA Network Open*.

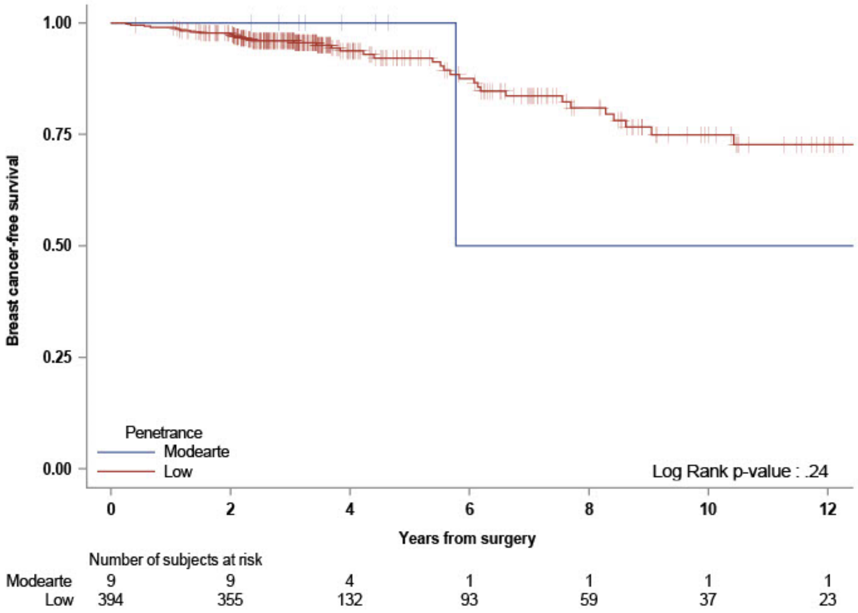

eFigure 2.

A

© 2026 Corson G et al. *JAMA Network Open*.

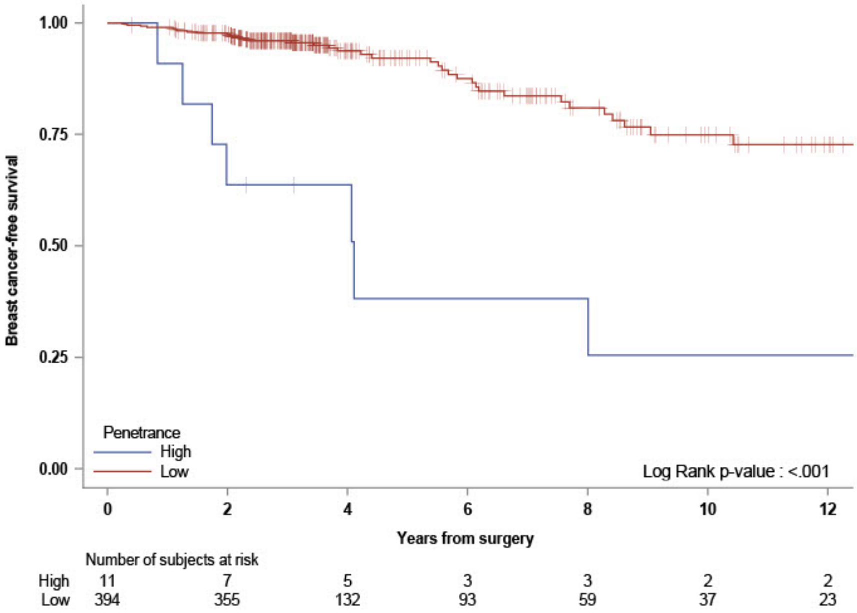

B

© 2026 Corson G et al. *JAMA Network Open*.

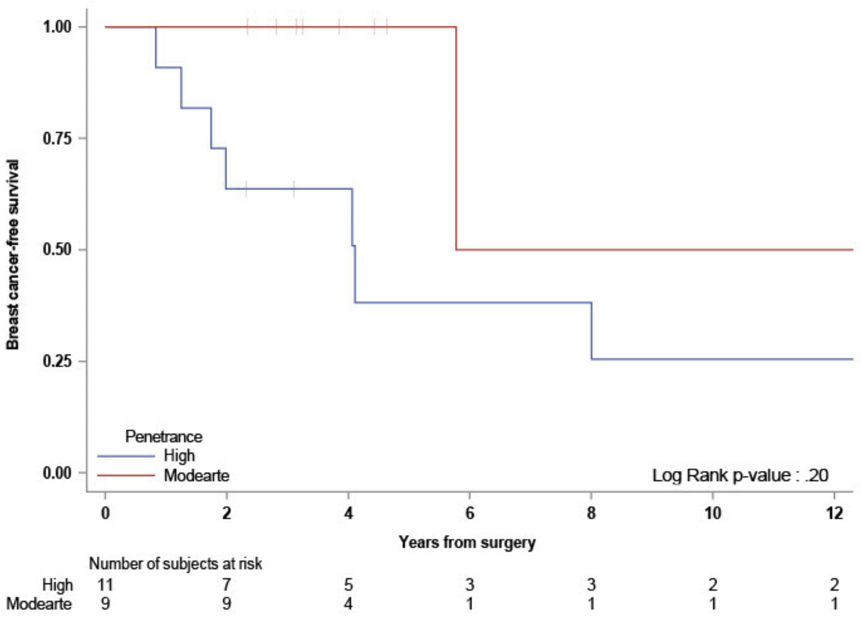

C

© 2026 Corson G et al. *JAMA Network Open*.
