# Supplementary material for: Germline Multigene Panel Testing in Women With Invasive Lobular Cancer
Source: JAMA Netw Open. 2026 Jul 8;9(7):e2621705. doi: 10.1001/jamanetworkopen.2026.21705 (PMC13347242; doi:10.1001/jamanetworkopen.2026.21705)
Supplement: Supplement 2. — Data Sharing Statement [file jamanetwopen-e2621705-s002.pdf]

Data Sharing Statement

Corso. Germline Multigene Panel Testing in Women With Invasive Lobular Cancer. *JAMA Netw Open*. Published July 08, 2026. doi:10.1001/jamanetworkopen.2026.21705

Data
Data available: No
